# eHealth Literacy and the Use of NHS 111 Online Urgent Care Service in England: Cross-Sectional Survey

**DOI:** 10.2196/50376

**Published:** 2024-06-04

**Authors:** Joanne Turnbull, Jane Prichard, Jennifer MacLellan, Catherine Pope

**Affiliations:** 1 School of Health Sciences University of Southampton Southampton United Kingdom; 2 Nuffield Department of Primary Care Health Sciences University of Oxford Oxford United Kingdom

**Keywords:** urgent care, digital health, access to health care, eHealth, health care system, COVID-19, urgent, emergency, health literacy, eHealth literacy, digital literacy, access, cross-sectional

## Abstract

**Background:**

Many health care systems have used digital technologies to support care delivery, a trend amplified by the COVID-19 pandemic. “Digital first” may exacerbate health inequalities due to variations in eHealth literacy. The relationship between eHealth literacy and web-based urgent care service use is unknown.

**Objective:**

This study aims to measure the association between eHealth literacy and the use of NHS (National Health Service) 111 online urgent care service.

**Methods:**

A cross-sectional sequential convenience sample survey was conducted with 2754 adults (October 2020-July 2021) **from** primary, urgent, or emergency care; third sector organizations; and the NHS 111 online website. The survey included the eHealth Literacy Questionnaire (eHLQ), questions about use, preferences for using NHS 111 online, and sociodemographic characteristics.

**Results:**

Across almost all dimensions of the eHLQ, NHS 111 online users had higher mean digital literacy scores than nonusers (*P*<.001). Four eHLQ dimensions were significant predictors of use, and the most highly significant dimensions were eHLQ1 (using technology to process health information) and eHLQ3 (ability to actively engage with digital services), with odds ratios (ORs) of 1.86 (95% CI 1.46-2.38) and 1.51 (95% CI 1.22-1.88), respectively. Respondents reporting a long-term health condition had lower eHLQ scores. People younger than 25 years (OR 3.24, 95% CI 1.87-5.62) and those with formal qualifications (OR 0.74, 95% CI 0.55-0.99) were more likely to use NHS 111 online. Users and nonusers were likely to use NHS 111 online for a range of symptoms, including chest pain symptoms (n=1743, 70.4%) or for illness in children (n=1117, 79%). The users of NHS 111 online were more likely to have also used other health services, particularly the 111 telephone service (*χ*_1_^2^=138.57; *P*<.001).

**Conclusions:**

These differences in eHealth literacy scores amplify perennial concerns about digital exclusion and access to care for those impacted by intersecting forms of disadvantage, including long-term illness. Although many appear willing to use NHS 111 online for a range of health scenarios, indicating broad acceptability, not all are able or likely to do this. Despite a policy ambition for NHS 111 online to substitute for other services, it appears to be used alongside other urgent care services and thus may not reduce demand.

## Introduction

### Background

“Digital first” as the central point of contact is increasingly being pursued in the delivery of a wide range of services, including health care [1]. The COVID-19 pandemic rapidly accelerated the use of apps, web-based digital technologies, and web-based triage in general practice and urgent care internationally [2] and in the United Kingdom [3]. Digital and telephone access are now core to primary [4,5], urgent, and emergency care provision in the UK NHS (National Health Service) [6,7], with a range of telephone and web-based services that triage and manage demand via e-consultation systems [6,8]. These systems typically offer urgent care call handling, web-based triage, and signposting to suitable services (eg, general practice, urgent care centers [UCCs], and emergency departments [EDs]) [9].

Digital health care offers the potential to improve the quality of patient care and provide timely and more convenient access to services [6]. They may also empower people to manage and maintain their own health [10]. Evidence suggests that participants often express high levels of satisfaction with web-based symptom checkers and assessment services [7]. However, important longstanding concerns that remain are socioeconomic and cultural factors [11], language difficulties, disability, and wider structural and technical infrastructure obstacles, which act as barriers to using, and benefitting from, digital access to health care [12]. Studies have shown that people from lower socioeconomic groups are typically less likely to use web-based information seeking [13] and symptom checkers [14]. Black or African American and Hispanic adults in the United States have been shown to be less likely to use technology for health-related purposes [15]. Conversely, younger and more highly educated people were more likely to use web-based triage and symptom checkers [7].

Accessing health care services via digital technologies predicates that people have sufficient knowledge, skills, resources, and motivation to access and use digital technologies to make decisions about a health problem [16]. The concept of eHealth literacy, which combines ideas about “health” and “health service” literacy (appreciation of symptoms and signs of illness and awareness of service provision) with digital literacy (ability to use digital technologies such as the web or smartphones) [16,17], has proved useful for examining this “digital divide” [11,18-20]. Studies using the eHealth literacy scale (eHEALS) [17] have demonstrated that lower eHealth literacy is associated with increased age [21,22], lower levels of education [23], lower socioeconomic status [22], and the presence of a long-term health condition (LTHC) [23]. Much of this literature has focused on eHealth literacy in relation to internet use for health information seeking rather than using symptom checkers or web-based triage. A survey using the eHealth Literacy Questionnaire (eHLQ) [24] reported that users who have digital access to health care services (eg, communicating with health professionals and accessing health-related information) scored higher on most dimensions of the scale [25]. However, little research has focused on eHealth literacy in the context of web-based urgent care triage and assessment.

### NHS 111 Online

The NHS 111 online urgent care service was launched in 2017 across the 4 nations of the United Kingdom and is an exemplar of a policy push to “digital first” that is not unique to the NHS. NHS 111 online was designed to augment the NHS 111 telephone triage and assessment service, which was launched in England in 2011 [3]. NHS 111 online is freely available 24 hours a day, giving access to web-based assessment and triage (via a smartphone, tablet, or computer) for people with urgent (nonemergency) care needs aged older than 5 years. The NHS 111 telephone and online services are both underpinned by a computer decision support software system. NHS 111 online users follow a tailored algorithm, answering questions about symptoms or health concerns. This results in an outcome that directs users to appropriate services (eg, emergency ambulance, ED, and general practice) or provides self-care advice. Where indicated, a call back from a health care professional may be offered. Facilities for booking arrival at an ED were more recently added [26]. In a single month (April 2024), 661,987 NHS 111 online sessions were completed: 64,754 (10%) resulted in an ambulance outcome; 73,366 (11%) emergency treatment; 283,808 (43%) primary care; 102,182 (15%), a prescription; 39,622 (6%) dental care; and 46,572 (7%) another service. Only 51,683 (8%) of calls resulted in self-management [27]. There is some expectation that NHS 111 online may help reduce or ameliorate demand for face-to-face urgent and emergency care services [3], but there is some evidence to suggest that NHS 111 online had little impact on the number of calls to the NHS 111 telephone service [28].

There is little research to date about eHealth literacy and the use of web-based triage and assessment urgent care services. Since NHS 111 online is used directly by patients and the public—without a call handler or clinical intermediary—this raises additional concerns about eHealth literacy and equity of access via digital services. It is unclear whether the potential benefits of urgent web-based health services, such as improving access to services and greater empowerment or self-management of own health [6,10], may be hindered by eHealth literacy. This study provides the first large-scale survey that aims to measure eHealth literacy and the help-seeking preferences of users and nonusers of NHS 111 online in the context of web-based urgent care use.

## Methods

### Study Design

A cross-sectional survey was conducted in England between October 2020 and July 2021, including periods when COVID-19 restrictions were in place. The survey included eHLQ, a validated 35-item 7-dimension questionnaire [28] used to explore individuals’ reported competencies, experiences, and interactions with technologies and services. The eHLQ consists of 7 dimensions: eHLQ1, using technology to process health information (5 items); eHLQ2, understanding of health concepts and language 5 items); eHLQ3, ability to actively engage with digital services (5 items); eHLQ4, feel safe and in control (5 items); eHLQ5, motivated to engage with digital services (5 items); eHLQ6, access to digital services that work (6 items); and eHLQ7, digital services that suit individual needs (4 items). The eHLQ was developed simultaneously in Danish and English using classical and modern test theory [28]. The instrument has been used in a range of countries and health care settings. Since its development, there have been several translations and cultural adaptations, and research indicates that the instrument is robust across a range of health care contexts [29-31].

The eHLQ is scored using a 4-point ordinal scale, from strongly disagree (1) to strongly agree (4). Each dimension contains between 4 and 6 items, with scores averaged to calculate each dimension. A higher mean score indicates a higher self-reported eHealth literacy score (a scale of 1 to 4). The highest score of 4 indicates individuals’ self-reported positive experiences and self-reported strengths and comfort with using digitized health services. The eHLQ does not include cut-off points or a benchmarking score for high or low eHealth literacy levels.

The survey also included questions about age, gender, educational attainment, employment status, and the presence of an LTHC. Respondents were asked if they had “a long-term condition or chronic disease” (eg, diabetes, chronic obstructive pulmonary disease, arthritis, and hypertension). As such, having an LTHC is defined by the respondents themselves. Educational attainment was aggregated to four levels: (1) no formal qualifications; (2) comprehensive or secondary school education equivalent—International Standard Classification of Education 2011 (ISCED-2011) levels 1 and 2; (3) further (short) education equivalent to ISCED levels 3, 4, and 5; and (4) higher education (medium and long equivalent to ISCED levels 6, 7, and 8) [32].

Additionally, 10 scenarios describing common presenting conditions or urgent care needs were used to explore preferences for using NHS 111 online. These scenarios were informed by data from our previous research [33] and developed in consultation with NHS Digital and patient and public representatives. Scenario preferences for using NHS 111 online were rated on a 5-point Likert scale from “very likely” to “very unlikely.” Respondents were also asked if they had ever previously used an urgent and emergency service (NHS 111 online, NHS 111 telephone service, UCC, general practice out-of-hours service, 999 emergency ambulance, and ED).

### Survey Sampling and Participants

Nonprobability sequential convenience sampling was a pragmatic choice to access people who had and had not previously used NHS 111 online. The sampling and recruitment strategy meant that it was not possible to calculate a response rate. Respondents were recruited via 24 primary care organizations, 7 urgent or emergency care settings, the NHS 111 online website, and 2 non-NHS third sector (charity) organizations. The small number of respondents from the charity sites (n=5 respondents) have been combined with primary care data in the analysis presented here. Potential respondents (aged 18 years or older) were identified sequentially by administrative or clinical staff at participating sites or organizations (eg, by reception staff at EDs, or general practice surgeries). General practices used an SMS text message mail out of the survey to eligible patients registered at their practice who had agreed to receive practice information via text message. Practices were asked to select a minimum of 100 random patients on their practice list that had consented to SMS mail outs. Some practices chose to sample more patients than 100 to increase recruitment numbers (practices sampled between an additional 1 and 135 patients per practice). EDs and UCCs invited attendees to their services to take part either by providing them with a web-based link to the survey or by offering the opportunity to complete the survey on a computer tablet in the waiting room (assisted, if necessary, by a research nurse). Sequential patients were offered a survey until a minimum of 50 participants had been recruited at each site. Patients in England who completed the NHS 111 online triage were offered a tailored hyperlink to complete the survey. Of 2754 valid responses, 1621 (58.9%) were recruited via primary care and charity settings, 626 (22.7%) through ED and UCC, and 507 (18.4%) via NHS 111 online.

### Patient and Public Involvement

A patient and public involvement (PPI) representative was on the project team and the study steering group and contributed to the design of the study and interpretation of the results. Additional PPI representatives (homeless health peer advocates of the charity Groundswell and members of the public from the Deep End Sheffield cluster PPI Group) took part in PPI events throughout the project, contributing to decisions about survey recruitment, helping to develop the scenario questions, and discussing the interpretation of results and how best to present information from the study for public audiences.

### Data Analysis

The analysis compared those who had previously used NHS 111 online at least once (users) and those who had not (nonusers). Descriptive categorical data are summarized and presented as frequency counts and percentages. Chi-square analysis was used to compare users and nonusers and whether they had ever used other urgent and emergency services and the likelihood of using NHS 111 online for the 10 health scenarios with previous use or nonuse of NHS 111 online. We created a binary variable of “likely” or “not likely” by removing the small number of neutral responses. Neutral responses accounted for 8%-16% of the data depending on the scenario. While this grouping loses some of the details of responses, it facilitates comparison. Analyses of the difference between users and nonusers were conducted using Bonferroni adjusted α levels of .007 per test (.05/7). Effect sizes are reported due to the large sample size (Φ correlation coefficient).

A secondary analysis was performed to assess the effects of age, gender, education, and use of NHS 111 online on eHealth literacy scores. Continuous data are presented as means (SDs). When comparing a continuous variable between 2 groups, 2-tailed *t* tests were applied. The mean eHLQ score for each dimension was compared for users and nonusers of NHS 111 online. Analysis of the difference in eHLQ scores was conducted using Bonferroni adjusted α levels of .007 per test (0.05/7). Due to the large sample size, effect sizes are reported (Hedges *g*).

Logistic regression was used to extend the univariate analysis outlined above to explore use versus nonuse of NHS 111 online. Logistic regression reports odds ratios (ORs) associated with each predictor value. The “enter” method (where all variables are entered into the model) was chosen so that all the chosen variables were entered into the model in a single step. Education was aggregated into a binary variable in the regression analysis since there was no strong association between eHLQ and education level (except that people with no formal qualifications had lower eHLQ scores compared to people with any level of qualification). The logistic regression model was examined for multicollinearity by examining tolerance, variance inflation factor, and variance of proportions.

We included respondents with incomplete data. Data for each analysis included all available values (case-by-case). In calculating the eHLQ dimensions, where more than 50% of the data were missing, a score was not calculated for that dimension and was excluded from the analysis.

### Ethical Considerations

This study involves human participants and ethical approval was granted for the study by the London Stanmore Research Ethics Authority (20/ LO/0294). Participants gave informed consent to participate in the study before taking part.

## Results

### Characteristics of Users and Nonusers of NHS 111 Online

Of 2754 valid respondents, 1617 (58.7%) had previously used NHS 111 online (“users”) and 1137 (41.3%) had not used NHS 111 online (“nonusers”). In total, 1745 (63.5%) of respondents were female, 1195 (43.6%) were aged between 45 and 64 years, and 1197 (44.2%) reported an LTHC (Table 1). More female participants reported using NHS 111 online, and the proportion of NHS 111 online users declined consistently with each increasing age and increased with the reported level of education. In total, there is a small difference in the proportion of people with a long-term or chronic condition who had used NHS 111 online compared to those who had not, 523 (46.7%) and 674 (42.4%), respectively.

**Table 1:** Characteristics of respondents by previous use of NHS 111 online (n=2754).

Characteristics			Nonuser, n (%)		User, n (%)		Total, n (%)
**Sex^a^**							
	Female	1000 (62)		745 (65.7)		1745 (63.5)	
	Male	606 (37.6)		373 (32.9)		979 (35.7)	
	Nonbinary or prefer not to say	6 (0.4)		16 (1.4)		22 (0.8)	
**Age group (years)**							
	18-24	55 (3.4)		104 (9.2)		159 (5.8)	
	25-34	149 (9.3)		182 (16)		331 (12.1)	
	35-44	245 (15.2)		203 (17.9)		448 (16.3)	
	45-54	328 (20.4)		240 (21.2)		568 (20.7)	
	55-64	405 (25.2)		222 (19.6)		627 (22.9)	
	65-74	321 (19)		139 (12.2)		460 (16.8)	
	≥75	106 (6.6)		44 (3.9)		150 (5.5)	
**Educational level**							
	No formal qualifications	233 (14.7)		83 (7.4)		316 (11.7)	
	Comprehensive school or General Certificate of Secondary Education or equivalent	327 (20.7)		213 (19)		540 (20)	
	Further education	420 (26.6)		334 (29.7)		754 (27.9)	
	Higher education (degree or higher)	601 (38)		493 (43.9)		1094 (40.5)	
**Long-term health condition^b^**							
	Yes	674 (42.4)		523 (46.7)		1197 (44.2)	
	No	916 (57.6)		597 (53.3)		1513 (55.8)	

^a^Sex: significant difference between male and female (*χ*_1_^2^=5.46; *P*=.02; Φ=0.05).

^b^Long-term health condition: significant difference between yes and no (*χ*_1_^2^=4.94; *P=*.03; Φ=0.04).

### eHealth Literacy

Across almost all dimensions of the eHLQ, as might be expected, NHS 111 online users had higher eHealth literacy (Table 2). Significant differences were observed for all dimensions except eHLQ4 (feel safe and in control) and eHLQ6 (access to digital services that work). Effect size calculations revealed that differences between users and nonusers were largest for the dimensions of eHLQ1 (using technology to process health information), eHLQ3 (ability to actively engage with digital services), and eHLQ5 (motivated to engage with digital services).

Respondents who reported having an LTHC tended to have lower eHLQ scores on some dimensions and yet were also more likely to have used NHS 111 online (Table 1). Further analysis identified that the subset of people with an LTHC who were nonusers of NHS 111 had the lowest eHLQ mean score for each dimension (Table 3). This difference was statistically significant for 5 dimensions when compared to users both with and without an LTHC but was not significant for eHLQ4 (feel safe and in control) and eHLQ6 (access to digital services that work).

**Table 2:** eHLQ^a^ dimensions by previous use of NHS 111 online.

Dimensions			Mean score (SD)		Mean difference (95% CI)		*t* value (*df*)		*P* value		Hedges *g*
**eHLQ1: using technology to process health information**					–0.22 (–0.26 to –0.17)		–9.00 (2677)		<.001		–0.35
	Nonuser (n=1565)	2.69 (0.62)									
	User (n=1114)	2.91 (0.60)									
**eHLQ2: understanding of health concepts and language**					–0.10 (–0.14 to –0.06)		–5.00 (2703)		<.001		–0.20
	Nonuser (n=1584)	2.96 (0.50)									
	User (n=1121)	3.06 (0.53)									
**eHLQ3: ability to actively engage with digital services**					–0.24 (–0.29 to –0.19)		–9.70 (2712)		<.001		–0.38
	Nonuser (n=1590)	2.91 (0.67)									
	User (n=1124)	3.15 (0.62)									
**eHLQ4: feel safe and in control**					0.003 (–0.04 to 0.05)		0.14 (2686)		.89		0.01
	Nonuser (n=1574)	2.96 (0.58)									
	User (n=1114)	2.95 (0.66)									
**eHLQ5: motivated to engage with digital services**					–0.17 (0.03 to –0.22)		–6.80 (2660)		<.001		–0.27
	Nonuser (n=1552)	2.51 (0.62)									
	User (n=1110)	2.68 (0.63)									
**eHLQ6: access to digital services that work**					–0.04 (0.02 to –0.09)		–1.82 (2728)		.068		–0.07
	Nonuser (n=1598)	2.71 (0.57)									
	User (n=1132)	2.75 (0.62)									
**eHLQ7: digital services that suit individual needs**					–0.09 (–0.15 to –0.04)		–3.50 (2668)		<.001		–0.09
	Nonuser (n=1559)	2.48 (0.67)									
	User (n=1111)	2.57 (0.70)									

^a^eHLQ: eHealth Literacy Questionnaire.

**Table 3:** eHLQ^a^ mean, SD, and mean difference for each dimension grouped by self-reported long-term health condition (yes or no) and NHS 111 online (nonuser or user).

Dimension		Mean score (SD)	Mean difference^b^		
			Nonuser (no)	User (yes)	User (no)
**eHLQ1**					
	Nonuser (yes)	2.63 (0.65)	–*0.11*^c^	–*0.24*	–*0.31*
	Nonuser (no)	2.74 (0.60)		–*0.14*	–*0.21*
	User (yes)	2.87 (0.62)			–0.07
	User (no)	2.94 (0.57)			
**eHLQ2**					
	Nonuser (yes)	2.94 (0.51)	–0.03	–*0.11*	–*0.12*
	Nonuser (no)	2.97 (0.48)		–*0.79*	–*0.09*
	User (yes)	3.05 (0.54)			–0.01
	User (no)	3.07 (0.52)			
**eHLQ3**					
	Nonuser (yes)	2.81 (0.72)	–*0.17*	–*0.29*	–*0.39*
	Nonuser (no)	2.98 (0.61)		–*0.12*	–*0.22*
	User (yes)	3.10 (0.66)			–*0.11*
	User (no)	3.21 (0.58)			
**eHLQ4**					
	Nonuser (yes)	2.96 (0.59)	0.01	0.02	–0.01
	Nonuser (no)	2.95 (0.57)		0.02	–0.02
	User (yes)	2.94 (0.67)			–0.03
	User (no)	2.97 (0.65)			
**eHLQ5**					
	Nonuser (yes)	2.47 (0.64)	–0.07	–*0.18*	–*0.24*
	Nonuser (no)	2.54 (0.61)		–*0.10*	–*0.17*
	User (yes)	2.64 (0.64)			–0.07
	User (No)	2.71 (0.62)			
**eHLQ6**					
	Nonuser (yes)	2.71 (0.58)	–0.01	–0.02	–0.07
	Nonuser (no)	2.72 (0.56)		–0.01	–0.06
	User (yes)	2.73 (0.63)			–0.05
	User (no)	2.78 (0.61)			
**eHLQ7**					
	Nonuser (yes)	2.41 (0.68)	–*0.12*	–0.08	–*0.24*
	Nonuser (no)	2.53 (0.65		0.04	–*0.11*
	User (yes)	2.49 (0.72)			–*0.15*
	User (no)	2.65 (0.67)			

^a^eHLQ: eHealth Literacy Questionnaire.

^b^Grouped by self-reported long-term health condition (yes or no).

^c^Italic formatting indicates significant differences between groups (*P*<.001).

### Use of Other Services In Addition to NHS 111 Online

The use of NHS 111 online is associated with increased previous use of other urgent and emergency services (Table 4; ie, if the respondent had ever used other urgent and emergency services). Notably, NHS 111 online users were likely to have also used the 111 telephone service.

**Table 4:** Previous use of urgent and emergency services by previous use of NHS 111 online.

Use of a service			Nonusers, n (%)	Users, n (%)		Chi-square (*df*=1)			*P* value		Φ	
**NHS 111 telephone**							138.57			<.001		0.22
	No (n=1211)	862 (53.3)		349 (30.7)								
	Yes (n=1543)	755 (46.7)		788 (69.3)								
**Urgent care center**							90.63			<.001		0.18
	No (n=1768)	1156 (71.5)		612 (53.8)								
	Yes (n=986)	461 (28.5)		525 (46.2)								
**General practice out-of-hours services**							86.08			<.001		0.18
	No (n=1941)	1249 (77.2)		692 (60.9)								
	Yes (n=813)	368 (22.8)		445 (39.1)								
**999 ambulance service**							23.12			<.001		0.09
	No (n=1835)	1136 (70.3)		699 (61.5)								
	Yes (n=919)	481 (29.7)		438 (38.5)								
**Emergency department**							33.35			<.001		0.11
	No (n=1428)	913 (56.6)		515 (45.3)								
	Yes (n=1326)	704 (43.5)		622 (54.7)								

### Scenarios Where NHS 111 Online Would Be Considered

There were 2 scenarios for which both users and nonusers reported they were especially likely to use NHS 111 online (Table 5); “young child with a temperature and crying” and “severe chest pain that goes away after a few minutes.” A sizeable proportion of nonusers reported that they might use NHS 111 online for seeking advice about young children (n=1117, 76.2%) or severe chest pain (n=1008, 69.3%). Nearly half of the nonusers also reported that they would be likely to use it for an itchy bite or sting (n=591, 42.5%), pain when urinating (n=696, 50.9%), and a headache for several hours (n=577, 43.3%).

**Table 5:** Likelihood of using NHS 111 online for different health scenarios.

Health scenarios and responses			Nonusers, n (%)		Users, n (%)		Chi-square (*df*=1)		*P* value		Φ
**Itchy bite or sting**							19.48		<.001		–0.09
	Likely (n=1097)	591 (42.5)		506 (51.6)							
	Unlikely (n=1275)	801 (57.5)		474 (48.4)							
**Young child’s temperature or crying**							16.51		<.001		–0.08
	Likely (n=1970)	1117 (76.2)		853 (83)							
	Unlikely (n=523)	348 (23.8)		175 (17)							
**Cough, cold, and sore throat**							1.25		.26		–0.02
	Likely (n=810)	462 (34.2)		348 (36.5)							
	Unlikely (n=1494)	888 (65.8)		606 (63.5)							
**Diarrhea or vomiting**							3.87		.05		–0.04
	Likely (n=972)	561 (40.8)		411 (44.9)							
	Unlikely (n=1319)	815 (59.2)		504 (55.1)							
**Scalded hand**							12.54		<.001		–0.07
	Likely (n=738)	399 (28.)		339 (34.8)							
	Unlikely (n=1658)	1024 (72)		634 (65.2)							
**Painful urinating**							31.42		<.001		–0.12
	Likely (n=1294)	696 (50.9)		598 (62.6)							
	Unlikely (n=1029)	672 (49.1)		357 (37.4)							
**Toothache >24 hours**							4.71		.03		–0.05
	Likely (n=894)	504 (36.3)		390 (40.7)							
	Unlikely (n=1454)	886 (63.7)		568 (59.3)							
**Headache for several hours**							3.35		.06		–0.04
	Likely (n=1014)	577 (43.1)		437 (46.9)							
	Unlikely (n=1257)	763 (56.9)		494 (53.1)							
**Tearful, not sleeping**							4.56		.03		–0.04
	Likely (n=620)	343 (24.9)		277 (28.9)							
	Unlikely (n=1713)	1032 (75.1)		681 (71.1)							
**Severe chest pain that subsides**							2.15		.14		–0.03
	Likely (n=1743)	1008 (69.3)		735 (72.1)							
	Unlikely (n=731)	446 (30.7)		285 (27.9)							

### Predicting Who Will Use NHS 111 Online

Logistic regression was used to predict the use (vs nonuse) of NHS 111 online for categorical variables such as age, gender, education, and LTHC and the mean scores for the 7 eHLQ continuous variables (Table 6). In the regression model, the reference group for age is the oldest group (≥75 years). For other variables, being female, no LTHC, and any qualification were the reference groups. Multicollinearity was tested in the model examining tolerance, the inverse of the tolerance, collinearity diagnostics, and the variance of proportions. Multicollinearity of greater than 0.5 occurred between dimensions 1 and 5 (0.63 for dimension 1 and 0.49 for dimension 5). Removing dimension 5 from the model improved the model fit slightly. Dimension 4 did not behave like the other dimension (there was little difference in this dimension between age, education, and LTHC), and so it was also removed from the model, providing a very small improvement in model fit. A total of 2534 respondents were included in the regression analysis, with 220 (8%) missing data either on at least 1 sociodemographic variable or eHLQ mean score. The model included 1055 respondents who had used NHS 111 online.

Age was a predictor of using NHS 111 online; people younger than 25 years (OR 3.24, 95% CI 1.87-5.62) and aged between 25 and 44 years (OR 2.35, 95% CI 1.47-3.75) were most likely to have used NHS 111 online. Although more women reported use of NHS 111 online, gender was not a significant predictor in the regression model. Education level was not a strong predictor of use, although those with formal qualifications were, perhaps unsurprisingly, more likely to report using NHS 111 online (95% CI 0.55-0.99). Respondents reporting LTHC had lower eHLQ scores and a subset of nonusers with an LTHC had the lowest eHLQ scores. Four eHLQ dimensions (eHLQ1, eHLQ2, eHLQ3, and eHLQ6) were significant predictors of NHS 111 online use, and most highly significant were dimensions eHLQ1 (using technology to process health information) and eHLQ3 (the ability to actively engage with digital services), with ORs of 1.86 (95% CI 1.46-2.38) and 1.51 (95% CI 1.22-1.88), respectively.

**Table 6:** Odds ratios for the likelihood of previous NHS 111 online use.

Characteristics		NHS 111 online users (n=1055)		Odds ratio (95% CI)		*P* value	
**Age (years)**							
	18-24 (n=132)	84	3.24 (1.87-5.62)		<.001		
	25-34 (n=304)	174	2.35 (1.47-3.75)		<.001		
	35-44 (n=418)	190	1.60 (1.02-2.49)		.04		
	45-54 (n=533)	229	1.44 (0.93-2.21)		.10		
	55-64 (n=583)	208	1.09 (0.71-1.67)		.69		
	65-74 (n=428)	130	0.90 (0.57-1.39)		.62		
	≥75 (n=136)	40	1.0				
**Sex**							.24
	Male (n=909)	348	0.90 (0.76-1.07)				
	Female (n=1625)	707	1.0				
**Education**							.04
	No qualifications (n=291)	77	0.74 (0.55-0.99)				
	Any qualifications (n=2243)	978	1.0				
**LTHC^a^**							<.001
	Yes (n=1124)	495	1.61 (1.35-1.93)				
	No (n=1410)	560	1.00				
**eHLQ^b^**							
	1 (n=2534)	1055	1.86 (1.46-2.38)		<.001		
	2 (n=2534)	1055	0.77 (0.60-0.99)		.04		
	3 (n=2534)	1055	1.51 (1.22-1.88)		<.001		
	6 (n=2534)	1055	0.78 (0.61-1.00)		.05		
	7 (n=2534)	1055	0.80 (0.64-1.00)		.06		

^a^LTHC: long-term health condition.

^b^eHLQ: eHealth Literacy Questionnaire.

## Discussion

### Principal Results and Comparison With Prior Work

Our findings are consistent with previous research, which shows that women [12,14,34] and younger people are more likely to use digital health services and that people with no formal qualifications are less likely to use NHS 111 online [11,12,35,36]. To our knowledge, this is the first time the eHLQ has been used to examine eHealth literacy in relation to the use of an urgent web-based health service (NHS 111 online). Despite relying on web-based data collection methods in some of our settings (due to COVID-19), we found clear differences in reported eHealth literacy between users and nonusers of NHS 111 online. This finding suggests that the digital divide may be even greater than our data indicate. Similar significant differences have been reported in other studies of users and nonusers of technologies, for example, in medical outpatients using the eHLQ [25] and the eHEALS instrument in a population of baby boomers and older adults seeking health information on the internet [34]. These eHealth literacy differences highlight the potential for digital exclusion and widening of health inequalities and warrant further investigation.

The survey showed that respondents who had an LTHC appeared more likely to use NHS 111 online compared to those without an LTHC. This is consistent with previous research [36] and might be used to argue that NHS 111 online is meeting a need for this group. However, our findings are more nuanced; respondents who reported having an LTHC tended to have lower eHLQ scores and the subset of respondents with very low eHLQ scores who reported having an LTHC had not used NHS 111 online. This apparent digital exclusion may be a cause for concern and a source of inequitable service provision.

People who currently use NHS 111 online appear to concurrently use a range of other urgent and emergency services. This may suggest that the web-based service is not a substitute for other services and does not seem to offer an alternative but an addition to the 111 telephone service in help seeking for urgent care. It is important to note that the survey question asked if respondents had *ever* used other urgent and emergency services, so we do not know if multiple services are used within a single episode of care (eg, using NHS 111 online in addition to other services such as NHS 111 telephone services), or whether different services are used at different time points for different reasons. The value and health benefits of NHS 111 online as an additional service are unclear but, given that one of the key functions of the service is to refer to and signpost to other services, it seems unlikely that NHS 111 online will reduce demand for other urgent and emergency care services.

Our survey showed that people would consider using NHS 111 online for a range of symptom presentations. It was worrying that significant numbers reported they might use NHS 111 online for potentially more serious chest pain symptoms. We asked 2 PPI groups to reflect on this finding and they suggested that the now ubiquitous use of internet searching might underlie this, ie, people experiencing a symptom for the first time would “Google it.” The use of NHS 111 online for help seeking about illness in children may be similarly problematic as this service is not intended use children younger than 5 years. The use of NHS 111 online for potentially more serious symptom scenarios or younger children may introduce unnecessary delays in getting help. More targeted information to clarify the scope of NHS 111 online and encourage greater awareness of appropriate use is necessary.

### Strengths and Limitations

This large cross-sectional survey is the first to report on the eHealth literacy of people using and not using urgent care triage and assess technology (NHS 111 online). We acknowledge the limitations of eHealth health literacy measures [37,38] and the problem of using self-reports to assess eHealth literacy, but the eHLQ has shown high construct validity, discriminant validity, and scale reliability [24,29,30]. The requirement to report the 7 dimensions separately adds analytical complexity compared to other measures, which offer a single digital literacy score (such as the eHEALS instrument) [16]. Our pragmatic recruitment strategy (designed to capture users and nonusers of NHS 111 online) meant that we were unable to calculate or estimate a response rate. Survey data collection was conducted primarily via the internet resulting inevitably in some bias toward digital literacy in our sample. Some population groups (such as older adults and people with very low educational attainment) may be underrepresented. Recruitment via general practices via text mail excluded those without access to text and those who had not consented to receiving text messages; again, this may disproportionally reduce the responses from some groups (eg, older people). Nonetheless, we have demonstrated differences in reported eHealth literacy and we contend that these are likely to underreport the digital divide, given that people with the lowest literacy and greatest barriers to access to digital technologies were less well represented in the survey.

Our data were collected from across England, including areas of deprivation and high health need. The survey took place during the COVID-19 pandemic 2020-2021 and health services will adjust coming out of the pandemic; however, NHS 111 online remains a core component of urgent care provision and demand management.

### Conclusions and Future Research

Our findings about eHealth literacy and use of NHS 111 online may not be surprising; younger and more educated people are more digitally literate and may be expected to be better able to use this urgent care service. However, we have identified important differences in reported eHealth literacy between users and nonusers of NHS 111 online, notably for those with LTHCs. Going forward, the NHS must ensure that “digital first” policies do not entrench or exacerbate health inequalities.

One of the hopes for NHS 111 online was that it would substitute for other services, such as telephone or face-to-face urgent and emergency care [3]. Our survey shows that NHS 111 online users were more likely to have used other NHS urgent and emergency care services in addition to using NHS 111 online, and they had higher cumulative use across these services compared to nonusers. The implications of this, both in terms of health outcomes and service costs, warrant further investigation.

Our survey also suggests that people who have not previously used NHS 111 online appear likely to consider using it for a wide range of health scenarios. Understanding this reservoir of demand and their eHealth literacy will be important as web-based services continue to develop.

## Abbreviations

ED: emergency department
eHLQ: eHealth Literacy Questionnaire
eHEALS: eHealth literacy scale
LTHC: long-term health condition
NHS: National Health Service
OR: odds ratio
PPI: patient and public involvement
UCC: urgent care center
